# Cervical Cancer Screening in Partly HPV Vaccinated Cohorts – A Cost-Effectiveness Analysis

**DOI:** 10.1371/journal.pone.0145548

**Published:** 2016-01-29

**Authors:** Steffie K. Naber, Suzette M. Matthijsse, Kirsten Rozemeijer, Corine Penning, Inge M. C. M. de Kok, Marjolein van Ballegooijen

**Affiliations:** Department of Public Health, Erasmus MC, University Medical Center Rotterdam, Rotterdam, the Netherlands; Rudjer Boskovic Institute, CROATIA

## Abstract

**Background:**

Vaccination against the oncogenic human papillomavirus (HPV) types 16 and 18 will reduce the prevalence of these types, thereby also reducing cervical cancer risk in unvaccinated women. This (measurable) herd effect will be limited at first, but is expected to increase over time. At a certain herd immunity level, tailoring screening to vaccination status may no longer be worth the additional effort. Moreover, uniform screening may be the only viable option. We therefore investigated at what level of herd immunity it is cost-effective to also reduce screening intensity in unvaccinated women.

**Methods:**

We used the MISCAN-Cervix model to determine the optimal screening strategy for a pre-vaccination population and for vaccinated women (~80% decreased risk), assuming a willingness-to-pay of €50,000 per quality-adjusted life year gained. We considered HPV testing, cytology testing and co-testing and varied the start age of screening, the screening interval and the number of lifetime screens. We then calculated the incremental cost-effectiveness ratio (ICER) of screening unvaccinated women with the strategy optimized to the pre-vaccination population as compared to with the strategy optimized to vaccinated women, assuming different herd immunity levels.

**Results:**

Primary HPV screening with cytology triage was the optimal strategy, with 8 lifetime screens for the pre-vaccination population and 3 for vaccinated women. The ICER of screening unvaccinated women 8 times instead of 3 was €28,085 in the absence of herd immunity. At around 50% herd immunity, the ICER reached €50,000.

**Conclusion:**

From a herd immunity level of 50% onwards, screening intensity based on the pre-vaccination risk level becomes cost-ineffective for unvaccinated women. Reducing the screening intensity of uniform screening may then be considered.

## Introduction

Infection with the human papillomavirus (HPV) has been identified as a necessary cause for cervical cancer [1]. Both the bivalent vaccine (targeting HPV-types 16/18), which is used in the Netherlands, and the quadrivalent vaccine (targeting HPV-types 6/11/16/18) are effective in preventing the two highly oncogenic types 16 and 18 [2, 3], that are found in roughly 80% of invasive cervical cancers [4]. Recently, a nonavalent vaccine has been approved [5], targeting seven oncogenic (and two non-oncogenic) HPV-types and thereby potentially preventing almost 90% of cervical cancers worldwide [6].

In the Netherlands, a catch-up campaign targeted all 13- to 16-year-old girls in 2009. Since 2010, all 12-year-old girls are offered vaccination. The three-dose vaccination coverage has steadily increased from 49% in the 1993 birth cohort to 61% in the 2000 birth cohort [7, 8]. In these partly vaccinated cohorts, the prevalence of HPV-16/18 infections is lower than in the pre-vaccination population. Therefore, unvaccinated women in those cohorts will be at lower risk for developing cervical cancer. While this indirect protective effect of vaccination, so-called herd immunity, will be limited at first, it is expected to increase over time [9]. It can be estimated by the percentage reduction in HPV-16/18 prevalence among unvaccinated women who were offered vaccination, as compared to totally unvaccinated cohorts. In the Netherlands, primary HPV screening will be implemented in 2016. From then, it could be relatively easy to monitor HPV-16/18 prevalence in unvaccinated women.

In many developed countries, vaccinated cohorts are approaching the start age of cervical cancer screening. Especially in settings where both vaccinated and unvaccinated women are well represented, it is unclear what screening strategy should be offered. In the youngest vaccinated cohorts (with limited herd immunity), vaccinated women are at much lower risk than unvaccinated women and screening based on vaccination status is likely more cost-effective than current uniform screening [10–13]. However, vaccinated women may not accept being offered less screening, solely because they adhered to vaccination guidelines. Screening based on vaccination status also requires the linkage of the screening invitational system with vaccination registries, which may not be (fully) possible in all settings.

As long as the follow-up of HPV vaccinated women in trials and population-based settings is not long enough to observe (statistical) differences in cervical cancer rates between vaccinated and unvaccinated cohorts, countries are reluctant to reduce the screening frequency. In the U.S., the same screening protocol is recommended for both vaccinated and unvaccinated women [14, 15]. European guidelines even state that HPV vaccines cannot replace or modify current routine cervical cancer screening protocols [16].

What is merely realized, is that women at reduced risk (due to either vaccination or herd immunity) could also be harmed by too intensive screening. These women will be offered more screening tests than needed, which increases their probability of being referred to the gynecologist in the absence of clinically relevant lesions. Women with abnormal cytology or HPV positive test results commonly experience fear, self-blame, distress and anxiety about cervical cancer, which reduces their quality of life [17, 18]. The ethical justification of continuing screening optimized to unvaccinated women instead of to those who adhered to vaccination guidelines, is therefore questionable. Moreover, it is probably very inefficient and cost-ineffective to do so. To avoid this inefficiency, screening should be optimized to vaccinated women as soon as unvaccinated women are substantially protected via herd immunity. We investigated at what level of herd immunity this would be justified for unvaccinated women.

## Materials and Methods

Using the MISCAN-Cervix model, we determined two optimal screening strategies: one for a pre-vaccination cohort, and one for a vaccinated cohort. To determine the level of herd immunity for which it would be cost-effective to replace the first strategy by the second, both strategies were applied to an unvaccinated cohort, assuming different levels of herd immunity.

### MISCAN-Cervix model

The MISCAN-Cervix model, which is described in more detail in the model profile (S1 Appendix), was used to estimate costs and effects of different screening strategies [19]. In all of the analyses presented here, we simulated a cohort of 1 million women. While none of these women were assumed to be affected by vaccination when determining the optimal screening strategy for the pre-vaccination population, all of them were assumed to be vaccinated when determining the optimal screening strategy for vaccinated women. Both these optimal strategies were then applied to unvaccinated women assuming various herd immunity levels.

A fraction of these women will acquire HPV-infections and/or develop cervical intraepithelial neoplasia (CIN) lesions. If these precursors progress to cervical cancer, women may die from the disease. If the population undergoes screening, the disease can be detected and treated in an earlier stage. As a result, cervical cancer death may be prevented or postponed.

The population at risk for cervical cancer was simulated based on demographic and hysterectomy data [20, 21]; mortality from other causes was estimated using the observed age-specific mortality in the Netherlands in 2013 [20]. The age-specific incidence of HPV-infections that progress to cervical cancer was calibrated to the age-specific incidence of cervical cancer, which was obtained from the Netherlands Cancer Registry (NCR) [22]. The age-specific incidence of pre-invasive lesions that do not progress to cervical cancer was calibrated so that the simulated detection rates of CIN lesions fit the observed detection rates in the Netherlands. These observed detection rates were obtained from the Dutch Network and National Database for Pathology (PALGA) for the period 2000–2007 [23]. The incidence of high-risk HPV-infections that do not progress to CIN was calibrated so that the simulated prevalence of all high-risk HPV-infections fits the observed high-risk HPV prevalence [24, 25].

In the model, disease is subdivided into seven sequential stages: high-risk HPV-infection, three pre-invasive stages (CIN grade I, II and III), and three invasive stages (International Federation of Gynecology and Obstetrics (FIGO) stages IA, IB and II+) [26]. Pre-invasive and FIGO IA stages can be diagnosed by screening only, because no symptoms will develop, whereas stages IB and II+ can also be clinically diagnosed. Because precursors are usually not progressive [27]; in the model, most HPV-infections will clear without ever resulting in neoplasia, and lesions in pre-invasive stages can regress spontaneously. In the hypothetical situation without competing other-cause mortality, undetected preclinical invasive neoplasia will always progress to clinical cancer. CIN grades I and II can develop in the absence of a high-risk HPV-infection; in that case the lesion will always regress. CIN grade III or worse can only develop if a high-risk HPV-infection is present [28].

### Screening policies

We simulated four different screening policies: (A) primary HPV screening with reflex cytology triage and cytology triage after six months (future Dutch screening program), (B) primary cytology with reflex HPV triage, (C) combined primary HPV and cytology (i.e. co-testing) with HPV triage after 12 months, and (D) primary cytology with cytology and HPV triage after six months and cytology triage after 18 months (current Dutch screening program). Policies (A) and (B) were already found to be cost-effective in case of no herd immunity [29]; policies (C) and (D) are included because of their resemblance with current practice in the U.S. and in the Netherlands, respectively.

### Screening schedules

Screening schedules differed by start age, screening interval and number of screens in a lifetime. Possible start ages were 25, 30, 35, 40 and 45 years. The screening interval varied from 5 to 20 years and the number of lifetime screens ranged from 1 to 12. Because screening women older than 80 years is not likely to be beneficial [30], all strategies ended at or before the age of 80. In this way, 312 screening schedules were created.

### Assumptions for screening and treatment

As we aimed to optimized screening for women who adhere to screening guidelines, we assumed full attendance in both primary screening and triage testing (S1 Table). The sensitivity of cytology (the probability that the result is at least atypical squamous cells of undetermined significance (ASCUS)) was assumed to be 40% for CIN grade I, 50% for CIN grade II and 75% for CIN grade III or cancer [31]. In the model calibration, the sensitivity of detecting at least high-grade squamous intraepithelial lesion (HSIL) was estimated to be 4% for CIN grade I, 18% for CIN grade II, 56% for CIN grade III and 60% for cervical cancer. The specificity of cytology was estimated at 97.6%. Based on the observed difference in CIN grade III or cancer detection rates between cytology and the HPV test, we assumed the sensitivity of the HPV test to be 85% for a high-risk HPV-infection [32]. Although contamination and cross-reactivity may cause HPV tests to produce positive results in the absence of high-risk HPV-infections, we assumed the specificity for the presence of HPV to be 100% and modelled a possible lack in specificity by including fast-clearing infections.

Detection of pre-invasive lesions and their associated management, including treatment if necessary, were assumed to lead to a 100% cure rate. A woman can, however, acquire new HPV-infections and develop CIN lesions after CIN treatment. For invasive cancer, we determined age-specific and stage-specific survival probabilities based on data from the NCR [33]. Since cancers detected by screening are found in an earlier stage than clinically diagnosed ones, women have a higher chance of survival. Using the NCR data, we estimated that if an invasive cancer is screen-detected, the probability to die from cervical cancer is reduced by 89.4%, 50% and 20% for FIGO stages IA, IB and II+, respectively [33].

### Assumptions for costs and utility losses

The estimated costs are based on a societal perspective, and are reported in 2013 euros (S2 Table). Screening costs include the costs for the invitational system and quality assurance, time and travel costs of the woman being screened, costs of smear taking, costs of evaluating the smear, costs of repeat tests after an inadequate test result, and costs of registration in PALGA. Diagnosis costs for women referred for colposcopy, treatment costs for detected pre-invasive lesions, treatment costs for invasive cervical cancer and costs of palliative care were derived from previous cost studies performed in the Netherlands [34]. A small (psychological) loss in quality of life was assumed for attending screening (including waiting for the result) and for being in triage (including attending follow-up screenings) [35]. Larger losses in quality of life were assumed for being diagnosed and treated for CIN or cancer, and for having a terminal stage of cervical cancer [36, 37]. Both costs and health effects were discounted with an annual rate of 3%.

### Assumptions for vaccination

We assumed the efficacy of the bivalent vaccine as observed in the PATRICIA trial^42,43^, which is 25.3% for HPV-infections without cytological abnormalities [38], and 35.0%, 54.8% and 93.2% for CIN grade I, II and III respectively (Table 1) [2]. As vaccination trials have not showed any waning in vaccine efficacy until now [39], the protection from vaccination was assumed to be lifelong. Due to limited follow-up of the trials, a reduction in cervical cancer incidence has not been observed yet. However, studies do give estimates of the type-specific reduction in HPV prevalence [40, 41]. In combination with the HPV-type distribution observed in cervical cancer cases in western Europe [4], the vaccine efficacy for cervical cancer was estimated at 83.8%. In this calculation we assumed that all cervical cancers are caused by a single oncogenic HPV-type, thereby avoiding overestimating the effect of the vaccine. We further assumed that all oncogenic types are equally likely to be co-infected with other oncogenic types, and decreased all type-specific HPV-positivity rates with the same percentage (6.6%) to account for multiple infections.

**Table 1:** Vaccination assumptions for base case analysis and sensitivity analyses.

	Vaccine type	Vaccine duration†	Vaccine efficacy				
			HPV-infections without CIN	CIN grade I	CIN grade II	CIN grade III	Cervical cancer
**Directly observed from PATRICIA trial (base case)**	Bivalent	Lifelong	25.3%	35.0%	54.8%	93.2%	83.8%¥
**Directly observed from FUTURE trial**	Quadrivalent	Lifelong	21.4%‡	29.7%	42.9%	45.5%	80.2%¥
Indirectly based on PATRICIA trial*	Bivalent	Lifelong	52.6%	34.4%	55.8%	62.5%	83.8%
Indirectly based on FUTURE trial*	Quadrivalent	Lifelong	42.6%	28.6%	50.6%	57.7%	80.2%

HPV = human papillomavirus; CIN = cervical intraepithelial neoplasia.

*Vaccine efficacy is calculated by combining the reduction in type-specific HPV-infections observed in the trial, with the HPV-type distribution observed in HPV-infections without cytological abnormalities (in the Netherlands) [43], and in CIN grade I, II, and III, and cervical cancer (in western Europe) [4].

†Trials do not (yet) show that vaccine efficacy wanes; we assumed that if it would, vaccine boosters would be offered.

¥Because the follow-up of the trials is too short to give (meaningful) estimates for cervical cancer, we used the estimates from the indirect approach.

‡Observed vaccine efficacy for high-risk HPV-infections combined with ASC-US (atypical squamous cells of undetermined significance), trial results do not include efficacy for high-risk HPV-infections only.

In the absence of herd immunity, unvaccinated women were assumed to have the cervical cancer risk as is currently observed in the Netherlands [42]. Full herd immunity was assumed to be equally effective as vaccination in preventing both HPV-infections, CIN lesions and cervical cancer. When the herd immunity level was assumed to be e.g. 25%, then 25% of the infections, lesions and cancers that would have been prevented by vaccination, were averted in unvaccinated women.

### Analyses and outcomes

For a pre-vaccination and a vaccinated cohort, we simulated the screening strategies described earlier and determined their discounted costs and effects as compared to no screening. For both cohorts, the optimal screening strategy was determined as follows. We first excluded all dominated screening strategies, i.e. those strategies that were more costly and less effective than (combinations of) other strategies. We then ranked the efficient strategies based on the number of quality-adjusted life years (QALYs) gained and calculated their incremental cost-effectiveness ratio (ICER), i.e. the additional costs per additional QALY gained compared to the next less effective, efficient strategy. For each cohort, the optimal screening strategy was then defined as the strategy with an ICER just below the willingness-to-pay threshold of €50,000 per QALY gained, which is a commonly used threshold in cost-effectiveness analyses for cervical cancer screening [29, 44].

The two optimal screening strategies were applied to unvaccinated women assuming herd immunity levels of 0%, 25%, 50%, 75% and 100%. For all these levels, the ICER of screening optimized to the pre-vaccination cohort as compared to screening optimized to the vaccinated cohort was calculated. If the ICER reached above €50,000 per QALY gained, screening optimized to the pre-vaccination risk level was no longer considered cost-effective for unvaccinated women.

### Sensitivity analyses

In the sensitivity analyses, we varied the following parameters.

### Vaccine efficacy

1. First, we used the vaccine efficacy from two randomized efficacy trials in which the quadrivalent vaccine was used (FUTURE I [45] and FUTURE II [46]). The efficacy found in these trials was lower than for the bivalent vaccine, i.e. 29.7%, 42.9% and 45.5% for CIN grade I, II and III lesions, respectively [47]. Because in these trials HPV testing was only used when cytological abnormalities were observed, the reduction in HPV-infections in women without cytological abnormalities is not known. Instead, we used the reduction in HPV-positive women with ASCUS, which was 21.4% [47]. Again, the efficacy for cervical cancer was estimated using the type-specific reduction in HPV prevalence [41, 48] and the HPV-type distribution in cervical cancer [4], which resulted in an estimate of 80.2%.
2. Second, we estimated the efficacy for all disease stages by using the type-specific reduction in HPV prevalence observed in the PATRICIA trial and the HPV-type distribution observed in the Netherlands (for HPV-infections without cytological abnormalities) [43] and in western Europe (for CIN lesions and cervical cancer) [4]. This resulted in an assumed vaccine efficacy of 52.6% for HPV-infections, and of 34.4%, 55.8% and 62.5% for CIN grade I, II and III respectively. For cervical cancer, the efficacy remained at its base case value of 83.8%.
3. Finally, this indirect approach of combining the type-specific reduction in HPV prevalence with the HPV-type distribution in HPV-infections, CIN lesions and cervical cancer was also used to determine the vaccine efficacy for the quadrivalent vaccine. The assumed vaccine efficacy was 42.6% for HPV-infections, 28.6%, 50.6%, 57.7% for CIN grade I, II and III respectively and 80.2% for cervical cancer.

#### Background risk for cervical cancer in unvaccinated women

Instead of assuming an equal background risk for vaccinated and unvaccinated women, we included two sensitivity analyses in which the background risk in unvaccinated women was assumed 50% higher and 50% lower than in vaccinated women.

## Results

### Base case analysis

For a pre-vaccination cohort, 6-yearly primary HPV screening in the age range 30–72 years is most cost-effective (S3 Table). This corresponds to 8 screens in a lifetime. The optimal strategy for vaccinated women is also primary HPV screening, but in a smaller age range (35–59 years) and with a longer interval (every 12 years), corresponding with 3 lifetime screens (S4 Table).

#### Health effects

As compared to screening 3 times, screening 8 times reduces cervical cancer deaths with 161 per 100,000 unvaccinated women in the absence of herd immunity, and with 28 in case of full herd immunity (Table 2). It thereby yields 388 and 34 more QALYs gained when assuming 0% and 100% herd immunity, respectively (Table 3). However, it also requires more screen tests, more referrals for colposcopy and more CIN treatments. For one additionally prevented death, the required additional number of referrals for colposcopy increased from 34 for 0% herd immunity to 118 for 100%.

**Table 2:** Undiscounted health effects for unvaccinated women of primary HPV screening at ages 30–72 every 6 years (optimal for unvaccinated women without herd immunity) and at ages 35–65 every 15 years (optimal for vaccinated women), as compared to no screening. For different levels of herd immunity, results are given per 100,000 unvaccinated women.

Herd immunity level	Screening strategy	# Primary screens	# Triage screens	# Referrals for colposcopy	# False-positive referrals (no CIN)	# CIN grade I	# CIN grade II	# CIN grade III	# Cases prevented	# Deaths prevented
0%	30–72, 6y	717,049	55,427	10,188	873	3,805	2,360	3,029	1,416	589
	35–59, 12y	277,073	20,127	4,718	271	1,479	1,014	1,782	982	423
25%	30–72, 6y	716,804	51,324	8,969	823	3,630	2,080	2,340	1,123	471
	35–59, 12y	277,153	18,450	4,085	257	1,421	889	1,383	776	338
50%	30–72, 6y	716,579	47,130	7,756	770	3,468	1,802	1,648	832	348
	35–59, 12y	277,233	16,752	3,447	242	1,357	765	985	579	248
75%	30–72, 6y	716,354	42,929	6,535	723	3,286	1,528	953	537	225
	35–59, 12y	277,308	15,054	2,803	229	1,290	632	589	372	161
100%*	30–72, 6y	716,113	38,739	5,472	678	3,121	1,254	252	230	98
	35–59, 12y	277,386	13,352	2,156	213	1,228	511	176	158	70

CIN = cervical intraepithelial neoplasia.

*We assume that with full herd immunity, unvaccinated women have the same cervical cancer risk as vaccinated women.

**Table 3:** Base case costs and QALYs gained as compared to no screening (both 3% discounted) of screening optimized to a pre-vaccinated cohort and of screening optimized to a vaccinated cohort, and incremental cost-effectiveness of the former strategy as compared to the latter. For different levels of herd immunity, results are given per 100,000 unvaccinated women.

Herd immunity level	Screening strategy			Costs	Incremental costs	QALYs gained	Incremental QALYs	ICER
	Age range	Interval	No. of screens					
0%	35–59	12y	3	€5,926,814		1,488		
	30–72	6y	8	€16,825,096	+€10,898,282	1,876	+388	€28,085
25%	35–59	12y	3	€5,136,318		1,184		
	30–72	6y	8	€16,064,406	+€10,928,088	1,495	+312	€35,042
50%	35–59	12y	3	€4,336,530		868		
	30–72	6y	8	€15,310,889	+€10,974,359	1,098	+231	€47,530
75%	35–59	12y	3	€3,539,526		556		
	30–72	6y	8	€14,556,455	+€11,016,928	698	+142	€77,541
100%*	35–59	12y	3	€2,720,635		231		
	30–72	6y	8	€13,816,140	+€11,095,505	265	+34	€322,234

QALY = quality-adjusted life year; ICER = incremental cost-effectiveness ratio

*We assume that with full herd immunity, unvaccinated women have the same cervical cancer risk as vaccinated women.

#### Costs and cost-effectiveness

**Fig 1:**
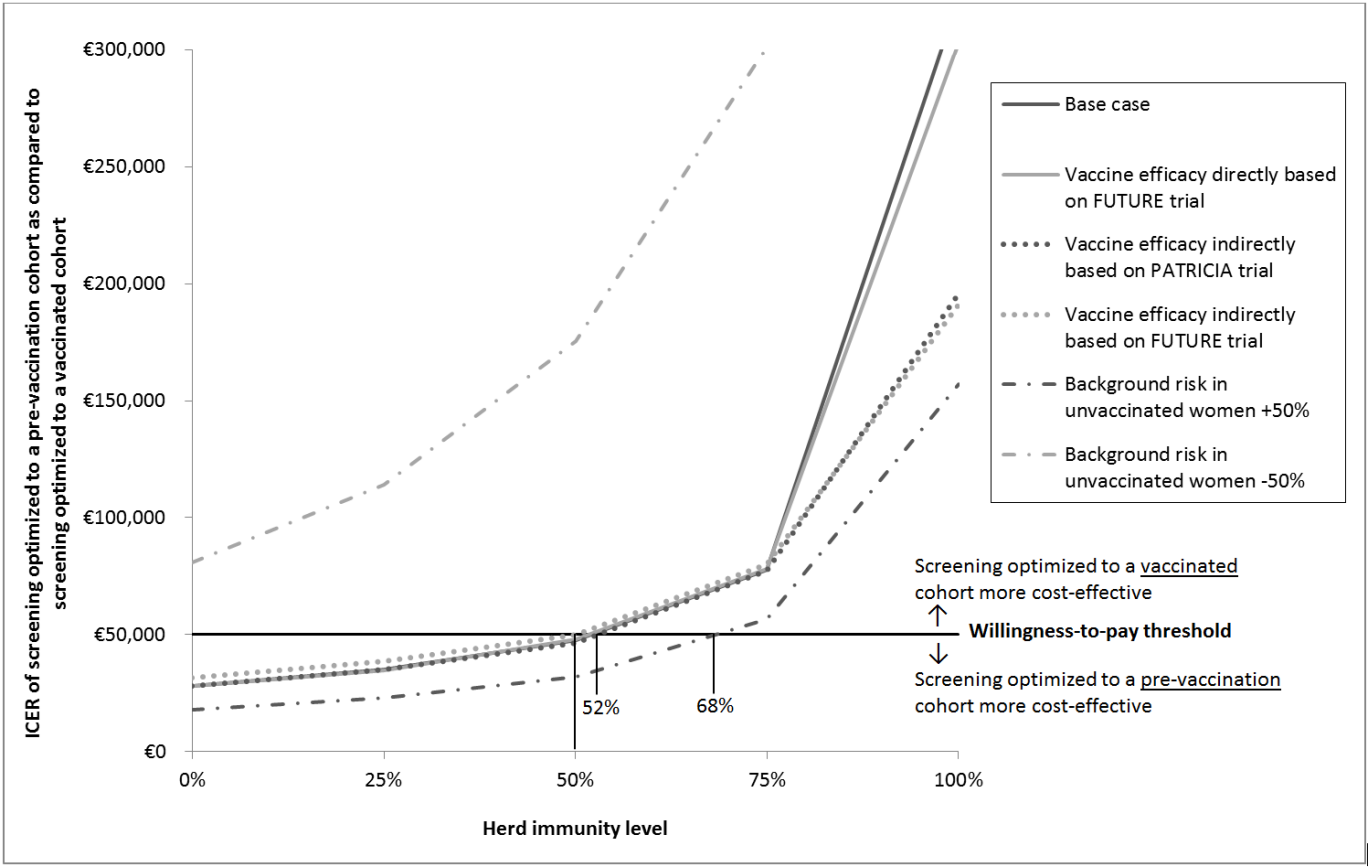
Incremental cost-effectiveness ratio (ICER) of screening optimized to a pre-vaccination cohort as compared to screening optimized to a vaccinated cohort, for unvaccinated women who benefit from different herd immunity levels, under both base case assumptions and sensitivity analyses.

Screening 8 times instead of 3 increases total costs with approximately €10.9 and €11.1 million assuming no and full herd immunity, respectively. Consequently, the ICER of screening 8 times instead of 3 increased from €28,085 per QALY gained in the absence of herd immunity to €35,042, €47,530, €77,541, and €322,234 for 25%, 50%, 75% and 100% herd immunity, respectively. From Fig 1, the estimated herd immunity level for which screening 8 times would cost approximately €50,000 per QALY gained when compared to screening 3 times, is 52%.

### Sensitivity analyses

When vaccine efficacy was calculated indirectly from the FUTURE trial, the optimal screening strategy for vaccinated women involved an additional screening round at age 71 (S5 Table). In all other sensitivity analyses, the optimal strategy for vaccinated women was unchanged (S6 and S7 Tables).

Similar to the base case analysis, the ICER of using the strategy optimized to the pre-vaccination cohort instead of to the vaccinated cohort, increased with increasing level of herd immunity (Table 4). In sensitivity analyses with different efficacy assumptions, screening optimized to the pre-vaccination population can be considered cost-effective as long as the herd immunity level is below 50%-52%. When unvaccinated women would have a 50% lower background risk for cervical cancer, screening can be optimized to vaccinated women, regardless of the herd immunity level. If instead, unvaccinated women have a 50% higher background risk, screening optimized to the pre-vaccination population should be continued until the herd immunity reaches above ~68%.

**Table 4:** Results sensitivity analyses: Incremental cost-effectiveness ratio of screening optimized to a pre-vaccination cohort, as compared to screening optimized to a vaccinated cohort.

Herd immunity level	Vaccine efficacy*			Background risk in unvaccinated women	
	Directly observed from FUTURE trial	Indirectly based on PATRICIA trial	Indirectly based on FUTURE trial	+50%	-50%
0%	€28,085	€28,085	€31,450	€17,828	€80,972
25%	€35,050	€34,675	€38,631	€22,950	€114,122
50%	€46,471	€48,097	€49,747	€31,998	€175,596
75%	€77,153	€78,139	€80,122	€56,390	€301,129
100%†	€195,881	€303,352	€191,000	€157,043	QALYs lost‡

QALYs = quality-adjusted life years.

*For vaccine efficacy assumptions, see Table 1.

†We assume that with full herd immunity, unvaccinated women have the same cervical cancer risk as vaccinated women.

‡For unvaccinated women at 50% reduced cervical cancer risk, QALYs were lost when screening was optimized to the pre-vaccination risk level instead of to the risk level in vaccinated women.

## Discussion

For both a pre-vaccination and a vaccinated cohort, primary HPV screening is more cost-effective than primary cytology or co-testing. The optimal number of lifetime screens varied from 8 for the pre-vaccination cohort, to only 3 for the vaccinated cohort. For unvaccinated women, the adverse effects and costs of screening become more important as the herd immunity level increases. Offering these women 8 instead of 3 lifetime screens incrementally required 34 colposcopy referrals per prevented death for 0% herd immunity, which increased to 118 referrals for 100% herd immunity. The ICER of screening 8 times instead of 3 increased from €28,085 per QALY gained in the absence of herd immunity to €322,234 at full herd immunity. Screening optimized to the risk level in vaccinated women becomes more cost-effective than screening optimized to the pre-vaccination risk level when the herd immunity reaches above 50%-55%.

To foresee whether and when the herd immunity will reach this level, countries need to monitor the HPV-16/18 prevalence in unvaccinated women, starting with a reliable pre-vaccination baseline measurement. A recent cross-sectional study among women aged 18–24 years in Australia, in whom vaccination coverage was 55%-74% for 1–3 doses [49], showed a reduction in HPV-16/18 prevalence of 93% and 35% in vaccinated and unvaccinated women, respectively, compared to the pre-vaccination prevalence [50]. From these early data, the estimated herd immunity level would equal (0.35 / 0.93 ≈) 38%.

We have not incorporated vaccination coverage as a separate parameter in our analyses, the reason for which is as follows. Vaccination coverage plays a role in two ways: first, it determines how many unvaccinated women there are (which is important when evaluating how to screen them), and second, it is one of the main determinants of herd immunity. Mathematical models have been created to estimate the level of herd immunity given vaccination coverage [51–53]. These models have been helpful in decision analyses concerning vaccination (also in boys), by estimating its indirect effect in the unvaccinated. However, when it comes to screening decisions that depend on current or near future herd immunity, it seems more appropriate to seek guidance from actual measurements (of HPV prevalence in the unvaccinated) than from model based predictions of herd immunity levels. Indeed, the exact relation between coverage and herd immunity will only become established based on such measurements.

The manuscript primarily focused on the effect of decreasing the screening frequency of uniform screening for unvaccinated women. For vaccinated women, this adjustment would be cost-effective by definition. Meanwhile, it is important to point out that the harms of screening the vaccinated 8 times instead of 3 were smaller than the life years gained (Table 3), meaning that unadjusted screening did not result in a net loss in health for vaccinated women.

We optimized the screening strategy to the pre-vaccination risk level and to the risk level in vaccinated women. For partly vaccinated cohorts, it could be beneficial to have a screening strategy that is a compromise of these two strategies. In fact, when ignoring the costs and efforts related to restructuring screening guidelines, it would likely be cost-effective to reduce the screening frequency gradually while the herd immunity level increases. Adjusting national screening guidelines every few years is not a very workable solution though. Likewise, it could be cost-effective to tailor screening to vaccination status. Our results have shown that as soon as the herd immunity level reaches 50%, then it is beneficial (in terms of cost-effectiveness) for unvaccinated women to replace screening optimized to the pre-vaccination risk level with screening optimized to the risk level in vaccinated women. If this already happens within a few years, then establishing tailored screening by e.g. developing a vaccination registry that is linked to the screening invitational system, may not be worthwhile. The (lack of) accumulation of herd immunity over time is crucial in deciding whether the establishment of tailored screening would be worth these additional efforts. We performed our analyses under the assumption that it is most realistic that countries will continue screening all women uniformly, and that a once-only adjustment is made as soon as this seems justified for unvaccinated women.

Notable limitations are the following. First, we assumed that the efficacy of the vaccine has a lifelong duration. Although until now, HPV vaccination trials have shown a sustained efficacy [2, 3], it is possible that the efficacy will wane in the future. If the protection would fade away and offering vaccination boosters would not be an option, then screening optimized to vaccinated women would probably be more intensive than in the current analyses, and unvaccinated women could be screened accordingly from a lower herd immunity level onwards. Second, as the follow-up of the vaccination trials is too limited to give (meaningful) estimates of the vaccine efficacy for cervical cancer, we had to estimate this efficacy indirectly. The decrease in CIN grade III lesions does indicate that the vaccine is likely to prevent clinically relevant lesions, and therefore also cancer [2, 47]. If the decrease in cervical cancer risk would be smaller than estimated, vaccinated women would also require more intensive screening, again meaning that unvaccinated women could be screened accordingly from a lower herd immunity level. Third, we assumed an equal background risk for vaccinated and unvaccinated women. Because reasons for refusing vaccination may vary widely (e.g. lack of knowledge about HPV, low perceived risk of infection, concerns about safety, religious values) [54], the background risk in unvaccinated women could both be higher or lower as compared to vaccinated women. In the sensitivity analyses we showed that even if the background risk in unvaccinated women would be 50% higher, then unvaccinated women could already be screened as vaccinated women from ~68% herd immunity onwards. Finally, we have not modeled the effects of the nonavalent vaccine, because its use is still limited compared to the bivalent and quadrivalent vaccine. If vaccination with this more potent vaccine would lead to a less intensive optimal screening strategy for vaccinated women, the herd immunity level at which unvaccinated women could be screened accordingly would be higher.

To our knowledge, this is the first study evaluating at what herd immunity level a once-only uniform (equal for vaccinated and unvaccinated women) screening adaptation becomes, considering risks, benefits and costs, an option. Because vaccinated women are approaching the age at which cervical cancer screening starts, the results of this study will be relevant in the near future. It shows, that as long as stepwise adjustment or dichotomized screening based on vaccination status are considered unfeasible, one may wait until the HPV-16/18 prevalence amongst unvaccinated women drops below 50% of the pre-vaccination level, before considering adjusting screening. Meanwhile, also the necessary evidence for a decrease in cervical cancer risk in vaccinated women should become available.

## Supporting Information

S1 Appendix MISCAN-Cervix model profile. (DOCX)

S1 Table Base case assumptions for screening. ASC-US = atypical squamous cells of undetermined significance; CIN = cervical intraepithelial neoplasia; HSIL = high-grade squamous intraepithelial lesion; HPV = human papillomavirus. *Potential false-positive HPV test results were modelled as HPV-infections with a short duration.
(DOCX)

S2 Table Base case assumptions for costs and utilities. HPV = human papillomavirus; CIN = cervical intraepithelial neoplasia; FIGO = International Federation of Gynecology and Obstetrics.Costs are in 2013 prices. €1.00 (£0.85; $1.37).
(DOCX)

S3 Table Cost-effective strategies for a pre-vaccination cohort under base case assumptions. QALY = quality-adjusted life year; ICER = incremental cost-effectiveness ratio; HPV = human papillomavirus.
(DOCX)

S4 Table Cost-effective strategies for a vaccinated cohort under base case assumptions. QALY = quality-adjusted life year; ICER = incremental cost-effectiveness ratio; HPV = human papillomavirus.
(DOCX)

S5 Table Cost-effective strategies for a vaccinated cohort when vaccine efficacy is indirectly based on the FUTURE trial. QALY = quality-adjusted life year; ICER = incremental cost-effectiveness ratio; HPV = human papillomavirus.
(DOCX)

S6 Table Cost-effective strategies for a vaccinated cohort when vaccine efficacy is directly observed from the FUTURE trial. QALY = quality-adjusted life year; ICER = incremental cost-effectiveness ratio; HPV = human papillomavirus.
(DOCX)

S7 Table Cost-effective strategies for a vaccinated cohort when vaccine efficacy is indirectly based on the PATRICIA trial. QALY = quality-adjusted life year; ICER = incremental cost-effectiveness ratio; HPV = human papillomavirus.
(DOCX)

## References

[1] Walboomers JM, Jacobs MV, Manos MM, Bosch FX, Kummer JA, Shah KV, et al Human papillomavirus is a necessary cause of invasive cervical cancer worldwide. J Pathol. 1999;189(1):12–9. Epub 1999/08/19. 10.1002/(SICI)1096-9896(199909)189:1<12::AID-PATH431>3.0.CO;2-F [pii] . 10451482

[2] Lehtinen M, Paavonen J, Wheeler CM, Jaisamrarn U, Garland SM, Castellsague X, et al Overall efficacy of HPV-16/18 AS04-adjuvanted vaccine against grade 3 or greater cervical intraepithelial neoplasia: 4-year end-of-study analysis of the randomised, double-blind PATRICIA trial. Lancet Oncol. 2012;13(1):89–99. Epub 2011/11/15. [pii] 10.1016/S1470-2045(11)70286-8 . 22075171

[3] Future I/II Study Group, Dillner J, Kjaer SK, Wheeler CM, Sigurdsson K, Iversen OE, et al Four year efficacy of prophylactic human papillomavirus quadrivalent vaccine against low grade cervical, vulvar, and vaginal intraepithelial neoplasia and anogenital warts: randomised controlled trial. BMJ. 2010;341:c3493 Epub 2010/07/22. 10.1136/bmj.c3493 20647284 PMC2907480

[4] Guan P, Howell-Jones R, Li N, Bruni L, de Sanjose S, Franceschi S, et al Human papillomavirus types in 115,789 HPV-positive women: a meta-analysis from cervical infection to cancer. Int J Cancer. 2012;131(10):2349–59. Epub 2012/02/11. 10.1002/ijc.27485 . 22323075

[5] U.S. Food and Drug Administration. FDA approves Gardasil 9 for prevention of certain cancers caused by five additional types of HPV Dec 10 2014. Available from: http://www.fda.gov/NewsEvents/Newsroom/PressAnnouncements/ucm426485.htm.

[6] Serrano B, Alemany L, Tous S, Bruni L, Clifford GM, Weiss T, et al Potential impact of a nine-valent vaccine in human papillomavirus related cervical disease. Infect Agent Cancer. 2012;7(1):38 Epub 2013/01/01. [pii] 10.1186/1750-9378-7-38 23273245 PMC3554470

[7] van 't Schurink-van 't Klooster TM, Kemmeren JM, Vermeer-de Bondt PE, Oostvogels B, Phaff TAJ, De Melker HE, et al. Human papillomavirus vaccination catch-up campaign in 2009 for girls born in 1993 to 1996 in the Netherlands. Bilthoven: National Institute for Public Health and the Environment, 2011 Contract No.: 210012001/2011.

[8] van Lier EA, Oomen PJ, Conyn-van Spaendonck MAE, Drijfhout IH, Zonnenberg-Hoff IF, De Melker HE. Immunisation coverage National Immunisation Programme in the Netherlands: Year of report 2015. Bilthoven: National Institute for Public Health and the Environment, 2015.

[9] Drolet M, Benard E, Boily MC, Ali H, Baandrup L, Bauer H, et al Population-level impact and herd effects following human papillomavirus vaccination programmes: a systematic review and meta-analysis. Lancet Infect Dis. 2015;15(5):565–80. Epub 2015/03/07. [pii] 10.1016/S1473-3099(14)71073-4 . 25744474 PMC5144106

[10] Diaz M, de Sanjose S, Ortendahl J, O'Shea M, Goldie SJ, Bosch FX, et al Cost-effectiveness of human papillomavirus vaccination and screening in Spain. Eur J Cancer. 2010;46(16):2973–85. Epub 2010/07/20. doi: S0959-8049(10)00532-0 [pii] 10.1016/j.ejca.2010.06.016 . 20638840

[11] Burger EA, Ortendahl JD, Sy S, Kristiansen IS, Kim JJ. Cost-effectiveness of cervical cancer screening with primary human papillomavirus testing in Norway. Br J Cancer. 2012;106(9):1571–8. Epub 2012/03/24. [pii] 10.1038/bjc.2012.94 22441643 PMC3341862

[12] Goldhaber-Fiebert JD, Stout NK, Salomon JA, Kuntz KM, Goldie SJ. Cost-effectiveness of cervical cancer screening with human papillomavirus DNA testing and HPV-16,18 vaccination. J Natl Cancer Inst. 2008;100(5):308–20. Epub 2008/03/04. [pii] 10.1093/jnci/djn019 18314477 PMC3099548

[13] Coupe VM, Bogaards JA, Meijer CJ, Berkhof J. Impact of vaccine protection against multiple HPV types on the cost-effectiveness of cervical screening. Vaccine. 2012;30(10):1813–22. Epub 2012/01/14. doi: S0264-410X(12)00002-3 [pii] 10.1016/j.vaccine.2012.01.001 . 22240341

[14] Saslow D, Solomon D, Lawson HW, Killackey M, Kulasingam SL, Cain J, et al American Cancer Society, American Society for Colposcopy and Cervical Pathology, and American Society for Clinical Pathology screening guidelines for the prevention and early detection of cervical cancer. Am J Clin Pathol. 2012;137(4):516–42. Epub 2012/03/21. doi: 137/4/516 [pii] 10.1309/AJCPTGD94EVRSJCG . 22431528

[15] Moyer VA, Force USPST. Screening for cervical cancer: U.S. Preventive Services Task Force recommendation statement. Ann Intern Med. 2012;156(12):880–91, W312. Epub 2012/06/20. doi: 1183214 [pii] 10.7326/0003-4819-156-12-201206190-00424 . 22711081

[16] European Centre for Disease Prevention and Control. Introduction of HPV vaccines in European Union countries—an update. Stockholm: 2012.

[17] Herzog TJ, Wright JD. The impact of cervical cancer on quality of life—the components and means for management. Gynecol Oncol. 2007;107(3):572–7. Epub 2007/10/30. doi: S0090-8258(07)00775-5 [pii] 10.1016/j.ygyno.2007.09.019 . 17963826

[18] Heinonen A, Tapper AM, Leminen A, Sintonen H, Roine RP. Health-related quality of life and perception of anxiety in women with abnormal cervical cytology referred for colposcopy: an observational study. Eur J Obstet Gynecol Reprod Biol. 2013;169(2):387–91. Epub 2013/05/07. doi: S0301-2115(13)00178-4 [pii] 10.1016/j.ejogrb.2013.03.033 . 23642971

[19] Habbema JD, van Oortmarssen GJ, Lubbe JT, van der Maas PJ. The MISCAN simulation program for the evaluation of screening for disease. Comput Methods Programs Biomed. 1985;20(1):79–93. Epub 1985/05/01. . 3849380 10.1016/0169-2607(85)90048-3

[20] Statline Database [Internet]. 2013.

[21] Hospital Diagnosis Statistics 1963–1985. Utrecht: SIG (Information Centre for Health Care), 1985.

[22] Netherlands Cancer Registry. Cervical cancer incidence by one-year age groups, 2000–2010. 2012.

[23] Casparie M, Tiebosch AT, Burger G, Blauwgeers H, van de Pol A, van Krieken JH, et al Pathology databanking and biobanking in The Netherlands, a central role for PALGA, the nationwide histopathology and cytopathology data network and archive. Cell Oncol. 2007;29(1):19–24. Epub 2007/04/13. . 17429138 10.1155/2007/971816 PMC4618410

[24] Bulkmans NW, Rozendaal L, Snijders PJ, Voorhorst FJ, Boeke AJ, Zandwijken GR, et al POBASCAM, a population-based randomized controlled trial for implementation of high-risk HPV testing in cervical screening: design, methods and baseline data of 44,102 women. Int J Cancer. 2004;110(1):94–101. Epub 2004/04/01. 10.1002/ijc.20076 . 15054873

[25] Lenselink CH, Melchers WJ, Quint WG, Hoebers AM, Hendriks JC, Massuger LF, et al Sexual behaviour and HPV infections in 18 to 29 year old women in the pre-vaccine era in the Netherlands. PLoS One. 2008;3(11):e3743 Epub 2008/11/18. 10.1371/journal.pone.0003743 19011683 PMC2581437

[26] Shepherd JH. Revised FIGO staging for gynaecological cancer. Br J Obstet Gynaecol. 1989;96(8):889–92. Epub 1989/08/01. . 2775686 10.1111/j.1471-0528.1989.tb03341.x

[27] Nobbenhuis MA, Helmerhorst TJ, van den Brule AJ, Rozendaal L, Voorhorst FJ, Bezemer PD, et al Cytological regression and clearance of high-risk human papillomavirus in women with an abnormal cervical smear. Lancet. 2001;358(9295):1782–3. Epub 2001/12/06. [pii] 10.1016/S0140-6736(01)06809-X . 11734239

[28] Stanley M. Pathology and epidemiology of HPV infection in females. Gynecol Oncol. 2010;117(2 Suppl):S5–10. Epub 2011/03/08. doi: S0090-8258(10)00087-9 [pii] 10.1016/j.ygyno.2010.01.024 . 20304221

[29] van Rosmalen J, de Kok IM, van Ballegooijen M. Cost-effectiveness of cervical cancer screening: cytology versus human papillomavirus DNA testing. BJOG. 2012;119(6):699–709. Epub 2012/01/19. 10.1111/j.1471-0528.2011.03228.x 22251259 PMC3489039

[30] Castanon A, Landy R, Cuzick J, Sasieni P. Cervical screening at age 50–64 years and the risk of cervical cancer at age 65 years and older: population-based case control study. PLoS Med. 2014;11(1):e1001585 Epub 2014/01/24. 10.1371/journal.pmed.1001585 PMEDICINE-D-13-02744 [pii]. 24453946 PMC3891624

[31] Berkhof J, de Bruijne MC, Zielinski GD, Meijer CJ. Natural history and screening model for high-risk human papillomavirus infection, neoplasia and cervical cancer in the Netherlands. Int J Cancer. 2005;115(2):268–75. Epub 2005/02/03. 10.1002/ijc.20846 . 15688404

[32] Rijkaart DC, Berkhof J, Rozendaal L, van Kemenade FJ, Bulkmans NW, Heideman DA, et al Human papillomavirus testing for the detection of high-grade cervical intraepithelial neoplasia and cancer: final results of the POBASCAM randomised controlled trial. Lancet Oncol. 2012;13(1):78–88. Epub 2011/12/20. [pii] 10.1016/S1470-2045(11)70296-0 . 22177579

[33] Netherlands Cancer Registry. Stage distribution and survival of cervical cancer 2012.

[34] van Ballegooijen M, Rebolj M, Essink-Bot ML, Meerding WJ, Berkers LM, Habbema JDF. De effecten en kosten van het bevolkingsonderzoek naar baarmoederhalskanker in Nederland na de herstructurering. Rotterdam: Erasmus MC, afdeling Maatschappelijke Gezondheidszorg, 2006.

[35] van Ballegooijen M. Effects and Costs of Cervical Cancer Screening. Rotterdam, the Netherlands: Erasmus University; 1998.

[36] Mandelblatt JS, Lawrence WF, Womack SM, Jacobson D, Yi B, Hwang YT, et al Benefits and costs of using HPV testing to screen for cervical cancer. JAMA. 2002;287(18):2372–81. Epub 2002/05/04. doi: joc11104 [pii]. . 11988058 10.1001/jama.287.18.2372

[37] Goldie SJ, Kohli M, Grima D, Weinstein MC, Wright TC, Bosch FX, et al Projected clinical benefits and cost-effectiveness of a human papillomavirus 16/18 vaccine. J Natl Cancer Inst. 2004;96(8):604–15. Epub 2004/04/22. . 15100338 10.1093/jnci/djh104

[38] Paavonen J, Naud P, Salmeron J, Wheeler CM, Chow SN, Apter D, et al Efficacy of human papillomavirus (HPV)-16/18 AS04-adjuvanted vaccine against cervical infection and precancer caused by oncogenic HPV types (PATRICIA): final analysis of a double-blind, randomised study in young women. Lancet. 2009;374(9686):301–14. Epub 2009/07/10. [pii] 10.1016/S0140-6736(09)61248-4 . 19586656

[39] Schiller JT, Castellsague X, Garland SM. A review of clinical trials of human papillomavirus prophylactic vaccines. Vaccine. 2012;30 Suppl 5:F123–38. Epub 2012/12/05. doi: S0264-410X(12)00951-6 [pii] 10.1016/j.vaccine.2012.04.108 . 23199956 PMC4636904

[40] Wheeler CM, Castellsague X, Garland SM, Szarewski A, Paavonen J, Naud P, et al Cross-protective efficacy of HPV-16/18 AS04-adjuvanted vaccine against cervical infection and precancer caused by non-vaccine oncogenic HPV types: 4-year end-of-study analysis of the randomised, double-blind PATRICIA trial. Lancet Oncol. 2012;13(1):100–10. Epub 2011/11/15. [pii] 10.1016/S1470-2045(11)70287-X . 22075170

[41] Malagon T, Drolet M, Boily MC, Franco EL, Jit M, Brisson J, et al Cross-protective efficacy of two human papillomavirus vaccines: a systematic review and meta-analysis. Lancet Infect Dis. 2012;12(10):781–9. Epub 2012/08/28. [pii] 10.1016/S1473-3099(12)70187-1 . 22920953

[42] www.cijfersoverkanker, Incidence, survival, and mortality of cervical cancer [Internet]. 2009.

[43] Coupe VM, Berkhof J, Bulkmans NW, Snijders PJ, Meijer CJ. Age-dependent prevalence of 14 high-risk HPV types in the Netherlands: implications for prophylactic vaccination and screening. Br J Cancer. 2008;98(3):646–51. Epub 2008/01/10. [pii] 10.1038/sj.bjc.6604162 18182990 PMC2243156

[44] Accetta G, Biggeri A, Carreras G, Lippi G, Carozzi FM, Confortini M, et al Is human papillomavirus screening preferable to current policies in vaccinated and unvaccinated women? A cost-effectiveness analysis. J Med Screen. 2010;17(4):181–9. Epub 2011/01/25. doi: 17/4/181 [pii] 10.1258/jms.2010.010019 . 21258128

[45] Garland SM, Hernandez-Avila M, Wheeler CM, Perez G, Harper DM, Leodolter S, et al Quadrivalent vaccine against human papillomavirus to prevent anogenital diseases. N Engl J Med. 2007;356(19):1928–43. Epub 2007/05/15. doi: 356/19/1928 [pii] 10.1056/NEJMoa061760 . 17494926

[46] Future II Study Group. Quadrivalent vaccine against human papillomavirus to prevent high-grade cervical lesions. N Engl J Med. 2007;356(19):1915–27. Epub 2007/05/15. doi: 356/19/1915 [pii] 10.1056/NEJMoa061741 . 17494925

[47] Munoz N, Kjaer SK, Sigurdsson K, Iversen OE, Hernandez-Avila M, Wheeler CM, et al Impact of human papillomavirus (HPV)-6/11/16/18 vaccine on all HPV-associated genital diseases in young women. J Natl Cancer Inst. 2010;102(5):325–39. Epub 2010/02/09. [pii] 10.1093/jnci/djp534 . 20139221

[48] Brown DR, Kjaer SK, Sigurdsson K, Iversen OE, Hernandez-Avila M, Wheeler CM, et al The impact of quadrivalent human papillomavirus (HPV; types 6, 11, 16, and 18) L1 virus-like particle vaccine on infection and disease due to oncogenic nonvaccine HPV types in generally HPV-naive women aged 16–26 years. J Infect Dis. 2009;199(7):926–35. Epub 2009/02/25. 10.1086/597307 . 19236279

[49] Brotherton JM, Murray SL, Hall MA, Andrewartha LK, Banks CA, Meijer D, et al Human papillomavirus vaccine coverage among female Australian adolescents: success of the school-based approach. Med J Aust. 2013;199(9):614–7. Epub 2013/11/05. 10.5694/mja13.10272 [pii]. . 24182228

[50] Tabrizi SN, Brotherton JM, Kaldor JM, Skinner SR, Liu B, Bateson D, et al Assessment of herd immunity and cross-protection after a human papillomavirus vaccination programme in Australia: a repeat cross-sectional study. Lancet Infect Dis. 2014;14(10):958–66. Epub 2014/08/12. [pii] 10.1016/S1473-3099(14)70841-2 . 25107680

[51] Brisson M, van de Velde N, Franco EL, Drolet M, Boily MC. Incremental impact of adding boys to current human papillomavirus vaccination programs: role of herd immunity. J Infect Dis. 2011;204(3):372–6. Epub 2011/07/12. [pii] 10.1093/infdis/jir285 . 21742835

[52] Elbasha EH, Dasbach EJ, Insinga RP. Model for assessing human papillomavirus vaccination strategies. Emerg Infect Dis. 2007;13(1):28–41. Epub 2007/03/21. 10.3201/eid1301.060438 17370513 PMC2725801

[53] Choi YH, Jit M, Gay N, Cox A, Garnett GP, Edmunds WJ. Transmission dynamic modelling of the impact of human papillomavirus vaccination in the United Kingdom. Vaccine. 2010;28(24):4091–102. Epub 2009/11/17. doi: S0264-410X(09)01485-6 [pii] 10.1016/j.vaccine.2009.09.125 . 19909831

[54] Dempsey AF, Abraham LM, Dalton V, Ruffin M. Understanding the reasons why mothers do or do not have their adolescent daughters vaccinated against human papillomavirus. Ann Epidemiol. 2009;19(8):531–8. Epub 2009/04/28. doi: S1047-2797(09)00083-0 [pii] 10.1016/j.annepidem.2009.03.011 19394865 PMC2880849

