# Supplementary material for: Cervical Cancer Screening in Partly HPV Vaccinated Cohorts – A Cost-Effectiveness Analysis
Source: PLoS One. 2016 Jan 29;11(1):e0145548. doi: 10.1371/journal.pone.0145548 (PMC4732771; doi:10.1371/journal.pone.0145548)
Supplement: S1 Appendix — (DOCX) [file pone.0145548.s001.docx]

# MISCAN-Cervix model profile

In this supplement, we describe the model inputs of the Microsimulation Screening Analysis (MISCAN) model for cervical cancer [[1](#_ENREF_1)]. This model can be used to assess the harms and benefits of different screening programs for cervical cancer, as well as human papillomavirus (HPV) vaccination. The model has been used previously for cost-effectiveness analyses of cervical cancer screening and HPV vaccination [[2-6](#_ENREF_2)].

## Model structure

Fig 1 shows the structure of MISCAN-Cervix. The model consists of the following 3 parts: demography, natural history, and screening. The results can be used to estimate the (cost-)effectiveness of different screening programs. The assumptions used in each of these parts are described below.

####
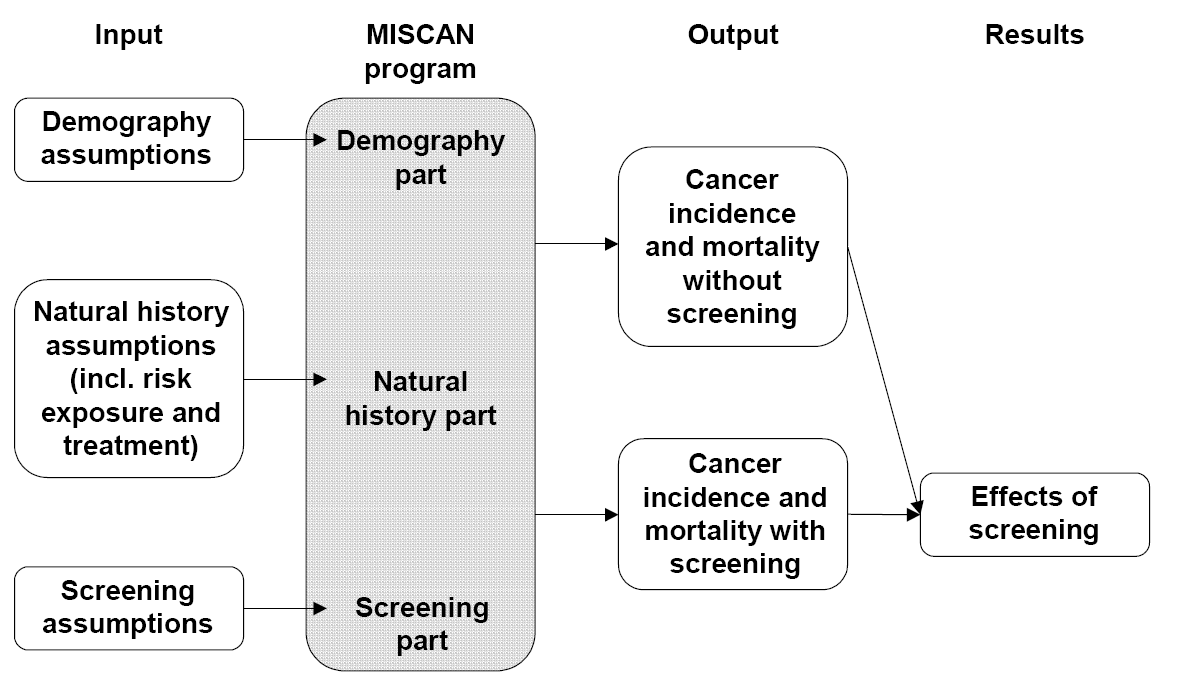


#### Fig 1. Structure of the MISCAN-Cervix model.

## Demography

The MISCAN model generates a simulated population. General characteristics of the simulated population (i.e. those not related to the disease) are based on demographic and hysterectomy data; mortality from other causes was estimated using the observed age-specific mortality in the Netherlands in 2011 [[7](#_ENREF_7),[8](#_ENREF_8)].

For each woman, a time of death from other causes (i.e. causes other than cervical cancer) is generated; this time of death is independent of the cervical cancer disease model. In the model, a woman’s lifetime cannot exceed 100 years. The time of death from other causes is generated using a life table for women from Statistics Netherlands [[8](#_ENREF_8)]. The assumed hysterectomy rates vary by age. These rates are based on data from Statistics Netherlands and Information Centre for Health Care and are presented in Table 1 [[9](#_ENREF_9),[10](#_ENREF_10)].

**Table 1. Model assumptions for the age-specific probability of having had a hysterectomy for reasons other than cervical cancer.**

| **Age** | **Cumulative probability of**  **having had a hysterectomy** |
| --- | --- |
| 20 | 0.0000 |
| 25 | 0.0002 |
| 30 | 0.0017 |
| 35 | 0.0076 |
| 40 | 0.0213 |
| 45 | 0.0432 |
| 50 | 0.0735 |
| 55 | 0.0916 |
| 60 | 0.1009 |
| 65 | 0.1102 |
| 70 | 0.1217 |
| 75 | 0.1330 |
| 80 | 0.1419 |
| 85 | 0.1468 |

Linear interpolation is used to determine the probability of having had a hysterectomy at intermediate ages.
Source: Information Centre for Health Care [[9](#_ENREF_9),[10](#_ENREF_10)].

## Natural history

During her lifetime, each woman has an age-specific risk of acquiring high-risk HPV infections (i.e. an infection caused by an HPV type that can cause cancer and that can be detected by the HPV test) and CIN lesions without a (detectable) high-risk HPV infection. Most HPV infections clear or regress naturally, some HPV infections can progress to CIN 1, CIN 2, CIN 3, cervical cancer, and death from cervical cancer.

The age-specific incidence of HPV infections that progress to cervical cancer was calibrated to the age-specific incidence of cervical cancer, which was obtained from the Netherlands Cancer Registry (NCR). The age-specific incidence of pre-invasive lesions that do not progress to cervical cancer was calibrated so that the simulated detection rates of CIN lesions fit the observed detection rates in the Netherlands. The observed detection rates were obtained from the Dutch Network and National Database for Pathology (PALGA) for the period 2000-2007. The incidence of high-risk HPV infections that do not progress to CIN was calibrated so that the simulated prevalence of all high-risk HPV infections fits the observed high-risk HPV prevalence [[11](#_ENREF_11),[12](#_ENREF_12)].

In MISCAN-Cervix, 6 disease pathways are distinguished. Each instance of these disease pathways represents an HPV infection or a ‘lesion’ (i.e. CIN of a certain grade or a stage of cervical cancer). Each disease pathway starts as either an HPV infection or as an HPV negative CIN 1 lesion. The natural history (i.e. in the situation without screening) of these 6 disease pathways is shown in Fig 2 and can be described as follows.

1. HPV infections that clear naturally without ever leading to CIN
2. HPV infections that progress to CIN 1 and then regress
3. HPV infections that progress to CIN 1 and CIN 2 and then regress
4. HPV infections that progress to CIN 1, CIN 2, and CIN 3 and then regress
5. HPV negative CIN 1 lesions that regress naturally or become HPV negative CIN 2 and then regress naturally
6. HPV infections that progress to CIN 1, CIN 2, CIN 3, preclinical FIGO 1A (micro-invasive) cervical cancer, and preclinical FIGO 1B cervical cancer. Preclinical FIGO 1B cervical cancer can either become clinically detected FIGO 1B cervical cancer or progress to preclinical FIGO 2+ cervical cancer and then to clinical FIGO 2+ cervical cancer. Clinically detected cervical cancer can progress to death from cervical cancer or remain in that state forever (if the woman is cured from cervical cancer).

A woman can acquire multiple lesions and HPV infections during her lifetime, and multiple lesions and HPV infections may be present at the same time. In each simulated life history (i.e. between ages 0 and 100), the number of lesions of each type follows a Poisson distribution. The annual probability of acquiring an HPV infection or CIN lesion is age-dependent and depicted in Figs 3A (regressive disease pathways) and 3B (progressive disease pathway). The transitions and sojourn times of the HPV infections or lesions are simulated based on a continuous-time semi-Markov process. The sojourn times of most states in the model have either an exponential or a Weibull probability distribution (Table 2).

In the model, women who do not have cervical cancer have an age-specific probability of getting a hysterectomy for reasons other than cervical cancer. A hysterectomy is assumed to remove all prevalent HPV infections and CIN lesions. Women with a hysterectomy will no longer acquire HPV infections or CIN lesions and are also no longer invited for screening tests.

**Fig 2. Schematic representation of the MISCAN model, with disease pathways A through F.**

**Fig 2 (continued). Schematic representation of the MISCAN-cervix model, with disease pathways A through F.**

Notes: There are six disease pathways (types A through F) in MISCAN. All lesions start as either an HPV infection without CIN (disease pathways A, B, C, D, and F) or as a CIN 1 lesion without HPV infection (disease pathway E). Cleared/regressed denotes the absence of CIN and HPV infection; CIN 0 denotes the absence of CIN and cervical cancer. All cervical cancer states are HPV positive. The arrows between the states show which types of transitions can occur; the numbers refer to the duration distributions shown in Table 5. In every state before death, a transition to “Other-cause death” can occur, and in every state before cancer, a transition to “Hysterectomy” can occur (connecting arrows not shown); in these cases, the transition applies to all HPV infections and CIN lesions of that person simultaneously.

**Table 2. Transitions and duration distributions used in MISCAN-Cervix.**

| **Transition number^a^** | **Disease pathway^a^** | **From state** | **To state** | **Probability of transition** | **Type of distribution** | **Mean duration (years)** | **Weibull shape parameter** |
| --- | --- | --- | --- | --- | --- | --- | --- |
| 1 | A | CIN 0 HPV+ | Cleared/regressed | 1 | Exponential | 1.0 | 1 |
| 2 | B, C, D | CIN 0 HPV+ | Cleared/regressed | 1 | Exponential | 1.0 | 1 |
| 3 | B, C, D, F | CIN 0 HPV+ | CIN 1 HPV+ | 1 | Exponential | 1.0 | 1 |
| 4 | B | CIN 1 HPV+ | CIN 1 HPV- | 0.4 | Exponential | 1.5 | 1 |
| 5 | B | CIN 1 HPV+ | CIN 0 HPV+ | 0.3 | Exponential | 1.5 | 1 |
| 6 | B | CIN 1 HPV+ | Cleared/regressed | 0.3 | Exponential | 1.5 | 1 |
| 7 | B | CIN 1 HPV- | Cleared/regressed | 1 | Exponential | 1.0 | 1 |
| 8 | E | CIN 1 HPV- | Cleared/regressed | 1 | Exponential | 1.5 | 1 |
| 9 | C, D, F | CIN 1 HPV+ | CIN 2 HPV+ | 1 | Exponential | 1.5 | 1 |
| 10 | C | CIN 2 HPV+ | CIN 2 HPV- | 0.4 | Exponential | 2.0 | 1 |
| 11 | C | CIN 2 HPV+ | CIN 0 HPV+ | 0.3 | Exponential | 2.0 | 1 |
| 12 | C | CIN 2 HPV+ | Cleared/regressed | 0.3 | Exponential | 2.0 | 1 |
| 13 | C | CIN 2 HPV- | Cleared/regressed | 1 | Exponential | 1.0 | 1 |
| 14 | D, F | CIN 2 HPV+ | CIN 3 HPV+ | 1 | Exponential | 2.0 | 1 |
| 15 | D | CIN 3 HPV+ | CIN 3 HPV- | 0.4 | Weibull | 3.1 | 1.67 |
| 16 | D | CIN 3 HPV+ | CIN 0 HPV+ | 0.3 | Weibull | 3.1 | 1.67 |
| 17 | D | CIN 3 HPV+ | Cleared/regressed | 0.3 | Weibull | 3.1 | 1.67 |
| 18 | D | CIN 3 HPV- | Cleared/regressed | 1 | Exponential | 1.0 | 1 |
| 19 | E | CIN 1 HPV- | CIN 2 HPV- | 1 | Exponential | 1.5 | 1 |
| 20 | E | CIN 2 HPV- | Cleared/regressed | 1 | Exponential | 2.0 | 1 |
| 21 | F | CIN 3 HPV+ | Preclinical FIGO 1A | 1 | Weibull | 11.8 | 1.67 |
| 22 | F | Preclinical FIGO 1A | Preclinical FIGO 1B | 1 | Exponential | 3.2 | 1 |
| 23 | F | Preclinical FIGO 1B | Preclinical FIGO 2+ | Age-specific^b^ | Exponential | 0.5 | 1 |
| 24 | F | Preclinical FIGO 1B | Clinical FIGO 1B | Age-specific^b^ | Exponential | 0.5 | 1 |
| 25 | F | Preclinical FIGO 2+ | Clinical FIGO 2+ | 1 | Exponential | 1.3 | 1 |
| 26 | F | Clinical FIGO 1B | Cervical cancer death | Age-specific^c^ | Piecewise uniform | Age-specific^d^ | - |
| 27 | F | Clinical FIGO 2+ | Cervical cancer death | Age-specific^c^ | Piecewise uniform | Age-specific^d^ | - |

CIN = cervical intraepithelial neoplasia; FIGO = International Federation of Gynecology and Obstetrics. ^a^See Fig 2. ^b^Transition probability depends on age; see Table 3A. ^c^Transition probability depends on age; see Table 3B. ^d^See Table 3C for the duration distribution.

**Fig 3A.** Annual probability of acquiring a regressive HPV infection or CIN lesion

**Fig 3B.** Annual probability of acquiring a progressive HPV infection

The assumptions for the probability and the duration of survival after a clinically detected (i.e. detected because of symptoms) cervical cancer are based on data from the NCR for the period 1989-2009. As these data include both adenocarcinoma and squamous cell carcinoma, the survival we estimated is a weighted average of these two types of cervical cancer. We assumed that all cervical cancer mortality occurs in the first 10 years after diagnosis. The assumed probability of long-term survival depends on age and stage (FIGO 1B or FIGO 2+); in the model, FIGO 1A cervical cancer cannot be clinically detected. Table 3A shows what percentage of clinically detected cancers is detected in stages FIGO 1B and FIGO 2+. The model assumptions for the long-term survival probabilities are shown in Table 3B and the assumed duration distributions are shown in Table 3C.

**Table 3A. Age-specific probability that cervical cancer is detected in stages FIGO 1B and FIGO 2+, given that it is clinically detected.**

|  | **Clinical detection in stage:** | |
| --- | --- | --- |
| **Age** | **FIGO 1B** | **FIGO 2+** |
| 0 | 25.4% | 74.6% |
| 25 | 25.4% | 74.6% |
| 40 | 35.0% | 65.0% |
| 55 | 61.4% | 38.6% |
| 70 | 75.4% | 24.6% |
| 100 | 75.4% | 24.6% |

FIGO = International Federation of Gynecology and Obstetrics.
Percentages in the table are estimated in the model calibration. Linear interpolation is used to determine the probabilities at intermediate ages.
 **Table 3B. Model assumptions for the age-specific probability that clinical FIGO 1B and FIGO 2+ cervical cancer will lead to death from cervical cancer (i.e. 100% - probability of long-term survival), in the absence of other-cause mortality.**

| **Age** | **Clinical FIGO 1B** | **Clinical FIGO 2+** |
| --- | --- | --- |
| 0 | 9.7% | 45.5% |
| 30 | 9.7% | 45.5% |
| 45 | 10.8% | 51.1% |
| 60 | 22.9% | 55.4% |
| 80 | 34.5% | 68.7% |
| 100 | 34.5% | 68.7% |

FIGO = International Federation of Gynecology and Obstetrics.
Linear interpolation is used to determine the probabilities at intermediate ages. Source: observed age-specific and stage-specific survival for the periods 1989-2002 and 2003-2009, obtained from the NCR. **Table 3C. Model assumptions for the duration distribution of clinical FIGO 1B and FIGO 2+ cervical cancer, if the transition to death from cervical cancer occurs.**

| **Years after detection** | **Clinical FIGO 1B** | **Clinical FIGO 2+** |
| --- | --- | --- |
| 1 | 10.4% | 37.6% |
| 2 | 36.5% | 64.6% |
| 3 | 47.9% | 78.1% |
| 4 | 61.5% | 84.5% |
| 5 | 78.3% | 88.5% |
| 6 | 84.4% | 90.5% |
| 7 | 90.3% | 93.3% |
| 8 | 93.1% | 96.4% |
| 10 | 100% | 100% |

FIGO = International Federation of Gynecology and Obstetrics.
The values in this table represent the percentages of cervical cancer deaths that occur within a given number of years after the moment of clinical diagnosis. It is assumed that no cervical cancer mortality occurs more than 10 years after clinical diagnosis. Source: observed age-specific and stage-specific survival for the periods 1989-2002 and 2003-2009, obtained from the NCR.

## Screening

Screening can change the life histories of women. In the current analysis, we considered screening strategies in which we varied:

- the start age: 25, 30, 35, 40, and 45 years
- the number of screens: 1, 2, …, 11, 12 screens
- the interval: 5, 6, …, 19, and 20 years
- the policy: (A) primary HPV screening with cytology triage (t=0 and t=6),

(B) primary cytology with HPV triage (t=0),

(C) primary co-testing with HPV triage (t=12), and

(D) primary cytology with cytology (t=6 and t=18) and HPV triage (t=6),

where t = the time in months since primary screening.

As we were interested in optimizing screening for those who adhere to screening guidelines, both the screening uptake and the compliance to triage testing and referrals to colposcopy were assumed to be 100%.

When an HPV test is applied, each HPV infection prevalent at the time of screening has a probability of producing a positive test (i.e. the sensitivity). If the HPV test is positive, cytological inspection determines whether the woman is referred to colposcopy or invited for cytological triage after 6 months (policy A). When the primary test is cytology, the woman is directly referred to colposcopy if the result is a high-grade squamous intraepithelial lesion (HSIL) or worse. For abnormal cytological results less than HSIL, an HPV test determines whether the woman is referred to colposcopy or sent back to the routine program, when following policy B. In policy D, these women are invited for another cytological test after 6 months. Again, if the result is HSIL or worse, women are referred to the gynecologist. For abnormal results less than HSIL, an HPV test is used to determine whether the woman is referred for colposcopy (positive HPV test) or invited for another cytological test after 12 months from then (negative HPV test). If the cytological result is normal, women are only invited for another cytological test after 12 months if the smear tests positive on the presence of high-risk HPV infections. For policy D, we assumed that all primary testing includes both cytology and HPV testing. Women with HSIL or worse or with ASCUS/LSIL combined with a positive HPV test are directly referred for colposcopy. Those with an ASCUS/LSIL result testing negative on HPV and those with a normal cytological result testing positive on HPV, are invited for another HPV test after 12 months. HPV-positive women are then referred for colposcopy, and HPV-negative women return to the routine program. For the assumed test characteristics of the HPV test and cytology, see Supplemental Table 1.

If a woman is referred to colposcopy, all prevalent CIN lesions are assumed to be diagnosed and successfully removed. HPV infections without CIN are not treated. For screen-detected cervical cancer, a stage-specific improvement (compared to the situation without screening) in the probability of cure is assumed.

### The effects of early detection on survival

In the model, detection of cervical cancer by screening prevents death from cervical cancer in some but not all cases. However, if death from cervical cancer is not prevented, the time of death from cervical cancer is not changed by screening.

For screen-detected invasive cancers, survival was modelled as a reduction in the risk of dying compared with that risk in the situation without screening, when the cancer would have become clinical. This improvement of prognosis (89.4%, 50% and 20% for FIGO 1A, 1B and 2+ respectively) was calibrated to reproduce recently observed stage specific survival given observed screening (NCR).

## Cost-effectiveness

For each simulated woman who is alive, MISCAN-Cervix can determine the state, which can be Normal, HPV infected, CIN 1, CIN 2, CIN 3, FIGO 1A, FIGO 1B, and FIGO 2+. A woman can have multiple HPV infections or CIN lesions at the same time. Her state is determined by the most severe disease stage present, using the order HPV infection, CIN 1, CIN 2, CIN 3, FIGO 1A, FIGO 1B, and FIGO 2+ cervical cancer. If no HPV infections or CIN lesions are present, the woman’s state is Normal.

The model produces the number of life years spent in each state as well as the number of certain events (e.g. screenings and cervical cancer diagnoses) in a lifetime. For each of these events, Supplemental Table 2 presents the amount of quality-adjusted time lost and the associated costs. To calculate the total disutility of a screening strategy, a sum is taken over all the numbers of events multiplied by their associated quality-adjusted time lost. A similar approach is used to determine the costs of a screening strategy.

In the current analysis, we optimized screening for a pre-vaccination cohort, and a vaccinated cohort. We calculated the costs and effects of all screening strategies mentioned earlier, and determined the optimal strategy based on a willingness-to-pay threshold of €50,000 per quality-adjusted life year (QALY) gained. We then compared the costs and effects of these two strategies in unvaccinated women who benefit from 0%, 25%, 50%, 75% and 100% herd-immunity, where 100% herd-immunity was assumed to be equally effective as vaccination. Both vaccination and herd-immunity only provided protection against high-risk HPV-types 16 and 18.

## References

1. Habbema JD, van Oortmarssen GJ, Lubbe JT, van der Maas PJ (1985) The MISCAN simulation program for the evaluation of screening for disease. Comput Methods Programs Biomed 20: 79-93.

2. de Kok IM, van Ballegooijen M, Habbema JD (2009) Cost-effectiveness analysis of human papillomavirus vaccination in the Netherlands. J Natl Cancer Inst 101: 1083-1092.

3. van den Akker-van Marle ME, van Ballegooijen M, van Oortmarssen GJ, Boer R, Habbema JD (2002) Cost-effectiveness of cervical cancer screening: comparison of screening policies. J Natl Cancer Inst 94: 193-204.

4. de Kok IM, van Rosmalen J, Dillner J, Arbyn M, Sasieni P, et al. (2012) Primary screening for human papillomavirus compared with cytology screening for cervical cancer in European settings: cost effectiveness analysis based on a Dutch microsimulation model. BMJ 344: e670.

5. van Rosmalen J, de Kok IM, van Ballegooijen M (2012) Cost-effectiveness of cervical cancer screening: cytology versus human papillomavirus DNA testing. BJOG 119: 699-709.

6. Rozemeijer K, de Kok IM, Naber SK, van Kemenade FJ, Penning C, et al. (2015) Offering Self-Sampling to Non-Attendees of Organized Primary HPV Screening: When Do Harms Outweigh the Benefits? Cancer Epidemiol Biomarkers Prev 24: 773-782.

7. (1985) Hospital Diagnosis Statistics 1963-1985. Utrecht: SIG (Information Centre for Health Care).

8. (2013) Statline Database. The Hague: Statistics Netherlands (CBS).

9. SIG (Information Centre for Health Care) (1985) Hospital Diagnosis Statistics 1963-1985. Utrecht: SIG.

10. CBS (netherlands Central Bureau of Statistics) (1994) Death by cause of death, age and sex 1950-1992. Voorburg.

11. Bulkmans NW, Rozendaal L, Snijders PJ, Voorhorst FJ, Boeke AJ, et al. (2004) POBASCAM, a population-based randomized controlled trial for implementation of high-risk HPV testing in cervical screening: design, methods and baseline data of 44,102 women. Int J Cancer 110: 94-101.

12. Lenselink CH, Melchers WJ, Quint WG, Hoebers AM, Hendriks JC, et al. (2008) Sexual behaviour and HPV infections in 18 to 29 year old women in the pre-vaccine era in the Netherlands. PLoS One 3: e3743.

### 
