# Supplementary material for: Cervical Cancer Screening in Partly HPV Vaccinated Cohorts – A Cost-Effectiveness Analysis
Source: PLoS One. 2016 Jan 29;11(1):e0145548. doi: 10.1371/journal.pone.0145548 (PMC4732771; doi:10.1371/journal.pone.0145548)
Supplement: S1 Table — ASC-US = atypical squamous cells of undetermined significance; CIN = cervical intraepithelial neoplasia; HSIL = high-grade squamous intraepithelial lesion; HPV = human papillomavirus. *Potential false-positive HPV test results were modelled as HPV-infections with a short duration. (DOCX) [file pone.0145548.s002.docx]

**S1 Table. Base case assumptions for screening.**

| **Parameter** | **Value** |
| --- | --- |
| **Attendance** | 100% |
| **Cytology** |  |
| *Probability of at least ASC-US for:* |  |
| CIN grade I | 40% |
| CIN grade II | 50% |
| CIN grade III or worse | 75% |
| *Probability of at least HSIL for:* |  |
| CIN grade I | 4% |
| CIN grade II | 18% |
| CIN grade III | 56% |
| Cervical cancer | 60% |
| Specificity (CIN grade I or worse) | 97.6% |
| **HPV test** |  |
| Sensitivity for high-risk HPV-infection | 85% |
| Specificity for high-risk HPV-infection | 100%* |

ASC-US = atypical squamous cells of undetermined significance; CIN = cervical intraepithelial neoplasia; HSIL = high-grade squamous intraepithelial lesion; HPV = human papillomavirus.

*Potential false-positive HPV test results were modelled as HPV-infections with a short duration.
