# Supplementary material for: Cervical Cancer Screening in Partly HPV Vaccinated Cohorts – A Cost-Effectiveness Analysis
Source: PLoS One. 2016 Jan 29;11(1):e0145548. doi: 10.1371/journal.pone.0145548 (PMC4732771; doi:10.1371/journal.pone.0145548)
Supplement: S2 Table — HPV = human papillomavirus; CIN = cervical intraepithelial neoplasia; FIGO = International Federation of Gynecology and Obstetrics.Costs are in 2013 prices. €1.00 (£0.85; $1.37). (DOCX) [file pone.0145548.s003.docx]

**S2 Table. Base case assumptions for costs and utilities.**

|  | **Costs (€)** | | **Utilities** | | | |
| --- | --- | --- | --- | --- | --- | --- |
|  |  | | Disutility | Duration | | Quality-adjusted time lost |
| **Invitation** |  | |  |  | |  |
|  | 4.91 | | **-** | **-** | | **-** |
| **Primary screening** |  | |  |  | |  |
| Cytology | 66.95 | | 0.005 | 2 weeks | | 2 hours |
| HPV-test | 63.63 | |  |  |  |  |
| Cytology + HPV-test | 96.32 | |  |  |  |  |
| **Reflex triage** |  | |  |  | |  |
| Cytology | 32.69 | | - | - | | - |
| HPV-test | 29.38 | |  |  |  |  |
| **Triage after 6, 12 or 18 months** |  |  |  |  |  |  |
| Cytology | 64.41 | | 0.005 | Time since last test | | Depends on interval |
| HPV-test | 61.10 | | 0.005 |  |  |  |
| **Diagnosis and treatment of pre-invasive stages** | | |  |  | |  |
| False-positive referral | 300 | | 0.005 | 0.5 year | | 22 hours |
| CIN grade I | 936 | | 0.03 | 0.5 year | | 6 days |
| CIN grade II | 1,386 | | 0.07 | 1 year | | 26 days |
| CIN grade III | 1,623 | | 0.07 | 1 year | | 26 days |
| **Diagnosis and treatment of cancer** | | |  |  | |  |
| FIGO IA | 5,314 | | 0.062 | 5 years | | 4 months |
| FIGO IB | 12,601 | | 0.062 | 5 years | | 4 months |
| FIGO II+ (screen-detected) | 12,420 | | 0.28 | 5 years | | 17 months |
| FIGO II+ (clinically detected) | 11,599 | | 0.28 | 5 years | | 17 months |
| **Terminal care** |  | |  |  | |  |
|  | 28,220 | | 0.740 | 1 year | | 9 months |

HPV = human papillomavirus; CIN = cervical intraepithelial neoplasia; FIGO = International Federation of Gynecology and Obstetrics.
Costs are in 2013 prices. €1.00 (£0.85; $1.37).
