# Supplementary material for: Cervical Cancer Screening in Partly HPV Vaccinated Cohorts – A Cost-Effectiveness Analysis
Source: PLoS One. 2016 Jan 29;11(1):e0145548. doi: 10.1371/journal.pone.0145548 (PMC4732771; doi:10.1371/journal.pone.0145548)
Supplement: S3 Table — QALY = quality-adjusted life year; ICER = incremental cost-effectiveness ratio; HPV = human papillomavirus. (DOCX) [file pone.0145548.s004.docx]

**S3 Table. Cost-effective strategies for a pre-vaccination cohort under base case assumptions.**

| **Strategy** | | | | **Cost-effectiveness (3% discounted)** | | |
| --- | --- | --- | --- | --- | --- | --- |
| **Policy** | **Age range** | **Interval** | **No. of screens** | **QALYs gained** | **Costs** | **ICER** |
| Primary HPV with cytology triage | 45 | - | 1 | 817 | €3,656 | - |
| Primary HPV with cytology triage | 40 - 59 | 19 | 2 | 1,192 | €595,896 | €1,851 |
| Primary HPV with cytology triage | 40 - 57 | 17 | 2 | 1,210 | €654,022 | €3,093 |
| Primary HPV with cytology triage | 40 - 66 | 13 | 3 | 1,355 | €1,225,940 | €3,971 |
| Primary HPV with cytology triage | 35 - 71 | 12 | 4 | 1,545 | €2,777,862 | €8,138 |
| Primary HPV with cytology triage | 35 - 65 | 10 | 4 | 1,602 | €3,247,901 | €8,226 |
| Primary HPV with cytology triage | 35 - 71 | 9 | 5 | 1,662 | €4,013,836 | €12,866 |
| Primary HPV with cytology triage | 35 - 75 | 8 | 6 | 1,706 | €4,888,675 | €19,898 |
| Primary HPV with cytology triage | 35 - 70 | 7 | 6 | 1,726 | €5,505,034 | €30,264 |
| Primary HPV with cytology triage | 30 - 72 | 7 | 7 | 1,820 | €8,797,780 | €35,292 |
| **Primary HPV with cytology triage** | **30 - 72** | **6** | **8** | **1,857** | **€10,423,560** | **€43,175** |
| Primary HPV with cytology triage | 30 - 78 | 6 | 9 | 1,865 | €10,849,457 | €55,738 |
| Primary HPV with cytology triage | 30 - 75 | 5 | 10 | 1,890 | €13,024,210 | €85,673 |
| Primary HPV with cytology triage | 30 - 80 | 5 | 11 | 1,893 | €13,420,746 | €138,221 |
| Primary HPV with cytology triage | 30 - 74 | 4 | 12 | 1,897 | €16,495,407 | €865,290 |

QALY = quality-adjusted life year; ICER = incremental cost-effectiveness ratio; HPV = human papillomavirus.
