# Supplementary material for: Cervical Cancer Screening in Partly HPV Vaccinated Cohorts – A Cost-Effectiveness Analysis
Source: PLoS One. 2016 Jan 29;11(1):e0145548. doi: 10.1371/journal.pone.0145548 (PMC4732771; doi:10.1371/journal.pone.0145548)
Supplement: S4 Table — QALY = quality-adjusted life year; ICER = incremental cost-effectiveness ratio; HPV = human papillomavirus. (DOCX) [file pone.0145548.s005.docx]

**S4 Table. Cost-effective strategies for a vaccinated cohort under base case assumptions.**

| **Strategy** | | | | **Cost-effectiveness (3% discounted)** | | |
| --- | --- | --- | --- | --- | --- | --- |
| **Policy** | **Age range** | **Interval** | **No. of screens** | **QALYs gained** | **Costs** | **ICER** |
| Primary HPV with cytology triage | 45 | - | 1 | 115 | €1,618,507 | - |
| Primary HPV with cytology triage | 40 | - | 1 | 137 | €2,005,709 | €17,306 |
| Primary HPV with cytology triage | 40 - 57 | 17 | 2 | 178 | €3,057,518 | €25,930 |
| Primary HPV with cytology triage | 40 - 55 | 15 | 2 | 181 | €3,143,604 | €29,183 |
| Primary HPV with cytology triage | 35 - 50 | 15 | 2 | 198 | €3,855,345 | €39,937 |
| Primary HPV with cytology triage | 35 - 65 | 15 | 3 | 217 | €4,656,004 | €42,850 |
| **Primary HPV with cytology triage** | **35 - 59** | **12** | **3** | **226** | **€5,081,409** | **€45,286** |
| Primary HPV with cytology triage | 35 - 55 | 10 | 3 | 233 | €5,408,214 | €52,823 |
| Primary HPV with cytology triage | 35 - 65 | 10 | 4 | 242 | €6,238,514 | €88,735 |
| Primary HPV with cytology triage | 35 - 67 | 8 | 5 | 252 | €7,594,621 | €138,443 |
| Primary HPV with cytology triage | 35 - 75 | 8 | 6 | 254 | €8,139,809 | €257,058 |
| Primary cytology with HPV triage | 30 - 72 | 6 | 8 | 262 | €13,056,727 | €593,680 |
| Primary cytology with HPV triage | 30 - 78 | 6 | 9 | 263 | €13,534,997 | €862,056 |
| Primary cytology with HPV triage | 30 - 75 | 5 | 10 | 263 | €15,697,980 | €5,376,727 |

QALY = quality-adjusted life year; ICER = incremental cost-effectiveness ratio; HPV = human papillomavirus.
