# Supplementary material for: Cervical Cancer Screening in Partly HPV Vaccinated Cohorts – A Cost-Effectiveness Analysis
Source: PLoS One. 2016 Jan 29;11(1):e0145548. doi: 10.1371/journal.pone.0145548 (PMC4732771; doi:10.1371/journal.pone.0145548)
Supplement: S5 Table — QALY = quality-adjusted life year; ICER = incremental cost-effectiveness ratio; HPV = human papillomavirus. (DOCX) [file pone.0145548.s006.docx]

**S5 Table. Cost-effective strategies for a vaccinated cohort when vaccine efficacy is indirectly based on the FUTURE trial.**

| **Strategy** | | | | **Cost-effectiveness (3% discounted)** | | |
| --- | --- | --- | --- | --- | --- | --- |
| **Policy** | **Age range** | **Interval** | **No. of screens** | **QALYs gained** | **Costs** | **ICER** |
| Primary HPV with cytology triage | 40 - 53 | 13 | 2 | 123 | €3,475,978 | - |
| Primary HPV with cytology triage | 40 - 66 | 13 | 3 | 147 | €4,199,297 | €29,996 |
| Primary HPV with cytology triage | 35 - 59 | 12 | 3 | 176 | €5,354,578 | €40,512 |
| **Primary HPV with cytology triage** | **35 - 71** | **12** | **4** | **189** | **€5,955,460** | **€47,493** |
| Primary HPV with cytology triage | 35 - 75 | 10 | 4 | 197 | €6,465,406 | €57,658 |
| Primary HPV with cytology triage | 35 - 71 | 9 | 5 | 209 | €7,359,065 | €74,476 |
| Primary HPV with cytology triage | 35 - 75 | 8 | 6 | 216 | €8,312,103 | €149,656 |
| Primary HPV with cytology triage | 30 - 78 | 8 | 7 | 229 | €10,955,327 | €203,002 |
| Primary HPV with cytology triage | 30 - 72 | 6 | 8 | 234 | €13,466,715 | €457,539 |
| Primary HPV with cytology triage | 30 - 78 | 6 | 9 | 235 | €13,922,136 | €639,319 |
| Primary cytology with HPV triage | 30 - 75 | 5 | 10 | 236 | €16,060,457 | €1,680,746 |

QALY = quality-adjusted life year; ICER = incremental cost-effectiveness ratio; HPV = human papillomavirus.
