# Supplementary material for: Cervical Cancer Screening in Partly HPV Vaccinated Cohorts – A Cost-Effectiveness Analysis
Source: PLoS One. 2016 Jan 29;11(1):e0145548. doi: 10.1371/journal.pone.0145548 (PMC4732771; doi:10.1371/journal.pone.0145548)
Supplement: S6 Table — QALY = quality-adjusted life year; ICER = incremental cost-effectiveness ratio; HPV = human papillomavirus. (DOCX) [file pone.0145548.s007.docx]

**S6 Table. Cost-effective strategies for a vaccinated cohort when vaccine efficacy is directly observed from the FUTURE trial.**

| **Strategy** | | | | **Cost-effectiveness (3% discounted)** | | |
| --- | --- | --- | --- | --- | --- | --- |
| **Policy** | **Age range** | **Interval** | **No. of screens** | **QALYs gained** | **Costs** | **ICER** |
| Primary HPV with cytology triage | 45 | - | 1 | 178 | €1,597,944 | - |
| Primary HPV with cytology triage | 40 | - | 1 | 216 | €1,980,213 | €10,678 |
| Primary HPV with cytology triage | 40 - 58 | 18 | 2 | 273 | €3,018,666 | €16,067 |
| Primary HPV with cytology triage | 40 - 54 | 14 | 2 | 280 | €3,103,658 | €25,127 |
| Primary HPV with cytology triage | 35 - 65 | 15 | 3 | 334 | €3,806,337 | €27,861 |
| Primary HPV with cytology triage | 35 - 61 | 13 | 3 | 341 | €4,596,828 | €44,804 |
| **Primary HPV with cytology triage** | **35 - 59** | **12** | **3** | **344** | **€5,016,822** | **€48,674** |
| Primary HPV with cytology triage | 35 - 71 | 12 | 4 | 355 | €5,339,470 | €52,364 |
| Primary HPV with cytology triage | 35 - 65 | 10 | 4 | 364 | €6,159,223 | €53,712 |
| Primary HPV with cytology triage | 35 - 71 | 9 | 5 | 378 | €7,498,097 | €67,009 |
| Primary HPV with cytology triage | 35 - 75 | 8 | 6 | 384 | €8,036,359 | €156,284 |
| Primary cytology with HPV triage | 30 - 78 | 6 | 9 | 395 | €13,361,537 | €480,256 |
| Primary cytology with HPV triage | 30 - 75 | 5 | 10 | 395 | €15,496,811 | €37,371,197 |

QALY = quality-adjusted life year; ICER = incremental cost-effectiveness ratio; HPV = human papillomavirus.
