# Supplementary material for: Cervical Cancer Screening in Partly HPV Vaccinated Cohorts – A Cost-Effectiveness Analysis
Source: PLoS One. 2016 Jan 29;11(1):e0145548. doi: 10.1371/journal.pone.0145548 (PMC4732771; doi:10.1371/journal.pone.0145548)
Supplement: S7 Table — QALY = quality-adjusted life year; ICER = incremental cost-effectiveness ratio; HPV = human papillomavirus. (DOCX) [file pone.0145548.s008.docx]

**S7 Table. Cost-effective strategies for a vaccinated cohort when vaccine efficacy is indirectly based on the PATRICIA trial.**

| **Strategy** | | | | **Cost-effectiveness (3% discounted)** | | |
| --- | --- | --- | --- | --- | --- | --- |
| **Policy** | **Age range** | **Interval** | **No. of screens** | **QALYs gained** | **Costs** | **ICER** |
| Primary HPV with cytology triage | 45 | - | 1 | 129 | 1,515,666 | - |
| Primary HPV with cytology triage | 40 | - | 1 | 152 | 1,908,685 | 17,332 |
| Primary HPV with cytology triage | 40 - 57 | 17 | 2 | 196 | 2,904,752 | 22,669 |
| Primary HPV with cytology triage | 40 - 55 | 15 | 2 | 198 | 2,990,397 | 49,102 |
| **Primary HPV with cytology triage** | **35 - 59** | **12** | **3** | **237** | **4,931,273** | **49,180** |
| Primary HPV with cytology triage | 35 - 55 | 10 | 3 | 243 | 5,243,447 | 56,753 |
| Primary HPV with cytology triage | 35 - 65 | 10 | 4 | 255 | 6,030,039 | 66,648 |
| Primary HPV with cytology triage | 35 - 75 | 10 | 5 | 259 | 6,543,607 | 131,391 |
| Primary HPV with cytology triage | 35 - 75 | 8 | 6 | 264 | 7,863,497 | 259,251 |
| Primary cytology with HPV triage | 30 - 78 | 6 | 9 | 269 | 13,401,036 | 1,028,756 |

QALY = quality-adjusted life year; ICER = incremental cost-effectiveness ratio; HPV = human papillomavirus.
